# Modelling the cost-effectiveness and budget impact of pay-for-performance programs in health care

**DOI:** 10.1186/s12913-024-11796-1

**Published:** 2024-11-27

**Authors:** Afschin Gandjour

**Affiliations:** Frankfurt School of Finance & Management, Adickesallee 32-34, Frankfurt am Main, 60322 Germany

**Keywords:** Health care, Pay for performance, Cost-effectiveness, Decision modeling

## Abstract

**Introduction:**

With an upward trend in adoption by industrialized nations, pay-for-performance (P4P) mechanisms are increasingly recognized for fostering quality improvement in healthcare. P4P programs conventionally reward providers with supplemental payments upon achieving predefined performance targets. This study aims to utilize decision modelling to determine the cost-effectiveness and maximum incentive levels of P4P programs.

**Methods:**

A decision model, grounded in previously published models exploring the cost-effectiveness of implementation programs, was developed. The model evaluates the cost-effectiveness of P4P programs for an exogenous reward size under varied reward mechanisms and also in scenarios with and without a budget or time constraint.

**Results:**

In instances where P4P programs do not substitute other healthcare programs, their incremental cost-effectiveness ratio (ICER) is deterministically set and ranges from treatment ICER to infinity, irrespective of the reward mechanism. With a budget or time constraint in play, the P4P program is incentivized up to the point where its population cost or ICER aligns with that of the program being replaced.

**Conclusion:**

The proposed decision model effectively calculates the cost-effectiveness and budget impact of P4P programs, accommodating various reward mechanisms and scenarios, both with and without budget or time constraints.

**Supplementary Information:**

The online version contains supplementary material available at 10.1186/s12913-024-11796-1.

## Introduction

Industrialized countries are increasingly adopting pay-for-performance (P4P) mechanisms to enhance quality improvement. P4P programs reward providers with additional payments (bonuses) for achieving stipulated performance targets or penalize them for not meeting health goals [1]. The growing interest in the P4P concept is underpinned by several factors [2]. A growing concern about healthcare quality has been highlighted by numerous studies that reveal significant gaps in provider compliance with evidence-based clinical practices [3]. Additionally, the higher frequency of medical errors than previously anticipated by many health care experts is also a notable concern. Another contributing factor is the steady rise in health care expenditures. Some decision-makers in healthcare hope that augmentations in clinical quality will lead to healthier patients and, ultimately, long-term cost savings - a perspective known as the business case for quality [4].

Various possible schemes for paying performance bonuses exist, which may depend on absolute, improved, or relative performance standards [1, 5, 6]. An absolute performance threshold holds providers accountable to an external or predetermined target standard, unrelated to the performance of other health providers. For instance, a bonus might only be paid if a provider’s average performance surpasses this standard, and payment typically occurs on a per-provider basis. However, health professionals working in areas of high socioeconomic deprivation might be disadvantaged [7] and could potentially relocate to other areas. Moreover, setting overly ambitious targets could discourage poorly performing providers, resulting in an overall lower average performance compared to when targets are less demanding [8]. Conversely, a reimbursement based on improved performance rewards only enhancements beyond existing performance (on a per-patient or per-provider basis). If a relative performance threshold is utilized, providers compete against each other, and only those “winning the tournament” are rewarded (typically on a per-provider basis). An advantage of a relative scheme over an absolute scheme is that the total amount of incentive payments can be calculated ex ante [9]. Nonetheless, when reimbursement is based on relative performance, the relative standing is beyond the provider’s control [10], which may cause a disincentive.

In addition to the aforementioned schemes, continuous reward systems are also prevalent, which do not employ a specific threshold but reward participants directly in proportion to performance [11]. Additionally, P4P programs can be tailored to individuals (health professionals) or organizations (teams) [11]. P4P programs may also incentivize single indicators or measures that span entire care pathways and can be based on either voluntary or mandatory participation [11]. Concerning the “evidence that would support the superiority of one incentive structure over another,” a recent review of international literature found “no clear” evidence [12]. Nevertheless, a few recommendations have been provided, such as a preference for absolute performance measures over relative ones, provided that absolute performance targets are differentiated by providers’ baseline performance.

Addressing quality issues, P4P may tackle misuse, underuse, or overuse. Underuse is defined as the failure to provide a healthcare service when it would have produced a favorable outcome for a patient [13]. Overuse occurs when a healthcare service is provided under circumstances in which its potential for harm exceeds the possible benefit [13]. Misuse takes place when an appropriate service has been selected but a preventable complication occurs, preventing a patient from receiving the full potential benefit of the service [13].

While several cost-effectiveness analyses, including modeling studies (e.g., [14–16]), have been conducted on P4P programs, the number of such studies remains limited [17, 18]. Moreover, even the available published cost-effectiveness analyses might be somewhat misleading as they may not account for potential substitution effects. Specifically, due to time or budget constraints faced by providers, incentivizing one intervention might inadvertently lead to the abandonment of another, potentially more effective, intervention. Consequently, the aim of this study is to introduce a decision model capable of analyzing the cost-effectiveness, budget impact, and health benefits of P4P programs, considering various reward mechanisms and both in scenarios with and without budget or time constraints.

## Methods

### Previous models

The P4P decision model developed in this study, beginning with Eq. 2 below, is constructed upon previously published decision models related to the cost-effectiveness of implementation programs [19–23]. The model, particularly the one published in 2010a, estimates the long-term incremental cost-effectiveness ratio (ICER) associated with altering physician behaviors through education. This model illustrates how reducing the underuse of healthcare services through education influences long-term cost-effectiveness. It can estimate the cost-effectiveness of any strategy aimed at changing physician behaviors, especially those targeting office physicians (e.g., educational outreach visits, lectures, audit, and feedback). Moreover, the model can assess cost-effectiveness from the perspectives of various potential initiators of change programs, such as payers or government entities. Only non-compliant physicians benefit from the educational activities. When educational measures span beyond one year or have an impact extending past that timeframe, the present discounted value of future costs and benefits is calculated. Consequently, the model attributes future costs and benefits to the year in which the educational measure is administered. The model, which compares incremental costs and effects of implementation versus non-implementation, is described by the following equation:

1 $$\:ICER=\frac{n_{\mathrm{im}}\times\:\Delta{c}_\text{im}}{du\times\:n_\text{ptpr}\times\:p_\text{dx}\times\:{\lambda}_\text{hp}\times\:\Delta{\lambda}_\text{hp}\times{co}_\text{pt}\times\Delta b_\text{tx}}+\frac{\Delta c_\text{tx}}{\Delta b_\text{tx}}.$$

In this equation, $\:{n}_{\text{i}\text{m}}$ denotes the number of implementation sessions, $\:\varDelta{c}_{\text{i}\text{m}}$ the cost of one implementation session inclusive of the costs of educator training, $\:\:du$ the effect duration of an implementation session in years, $\:{n}_{\text{p}\text{t}\text{p}\text{r}\:}$ the average number of patients per practice. Furthermore, $\:{p}_{\text{d}\text{x}}$ is the disease prevalence in the target population, ${\lambda}_{\text{h}\text{p}}$ the proportion of patients impacted by a quality of care deficit due to non-compliance of physicians, $\:\:{\varDelta\lambda}_{\text{h}\text{p}}$ the relative change in $\lambda_{\mathrm{hp}}$ due to an implementation session, and $co_{\text{pt}}$ the patient compliance rate – defined as the acceptance rate of initiating drug treatment and adherence to the regimen. Additionally, $\:{\varDelta{b}}_{\text{t}\text{x}}$ represents the per-patient incremental health benefit from treatment, while $\:\varDelta{c}_{\text{t}\text{x}}$ is the per-patient incremental treatment cost, encompassing discounted downstream costs and savings.

It is crucial to note that the product in the numerator of the first term reflects multiple payments over time. The $\:\varDelta{c}_{\text{i}\text{m}}$ may or may not encompass the opportunity cost of physicians’ time, depending on whether this cost is modeled separately. Moreover, the equation presupposes that $\:{\lambda}_{\text{h}\text{p}}$ and $\:{\varDelta\lambda}_{\text{h}\text{p}}$ are independent, implying that an increase in the number of implementation sessions or the intensity of implementation leads to diminishing returns in quality improvement.

### P4P no-substitution model

#### ICER calculation

Building on the preceding section, we introduce our P4P model. Consistently, we adopt a payer’s perspective. Although we particularly reference P4P programs for physicians, the results are also applicable to other health professionals and teams. Initially, we explore the scenario where increased physician effort does not substitute other physician services, assuming no time or budget constraint.

P4P programs founded on continuous performance reward each treated patient, while those based on improved performance reward each additional patient treated or an enhancement in the average treatment rate across patients. Contrasting with an improved performance scheme, P4P programs based on either absolute or relative performance mechanisms do not reward enhanced performance in providers who remain below a set threshold. However, for all reward schemes, implementation costs are incurred at the provider level, encompassing communication, administration, and data collection costs, along with the expense of the behavior change itself [11]. From a payer’s perspective, these costs are not relevant, since payers do not directly reimburse providers for incurring implementation costs in a P4P program in the manner of cost-plus reimbursement. Therefore, from a payer’s viewpoint, calculating the ICER of a P4P program does not necessitate modeling implementation costs or adopting the assumptions about implementation returns implied by Eq. 1. Instead, an ICER calculation requires modeling the size of the reward per patient treated, which, to a payer, is the cost of a reward and represents a form of implementation cost. In instances of an improved or continuous performance standard, the reward is always issued when an additional patient is treated. In this situation, the ICER calculation presented in Eq. 1 simplifies to:

2 $$\:ICER=\frac{n_{\text{P}4\text{P}}\times\Delta c_{\text{P}4\text{P}}}{\Delta b_\text{tx}}+\frac{\Delta c_\text{tx}}{\Delta b_\text{tx}},$$

where $\:{n}_{\text{P}4\text{P}}$ refers to the total number of P4P payments received per patient treated. $\:\varDelta{c}_{\text{P}4\text{P}}$ denotes the cost of one implementation session, which in the case of a P4P program corresponds to the size of a reward per patient treated and is exogenous (note that $\Delta c_\text{tx}$ and $\Delta b_{\text{tx}}$ refer to the cost and benefit per patient treated, respectively). It should be noted that both $\:{n}_{\text{P}4\text{P}}$ and $\:\varDelta{c}_{\text{P}4\text{P}}$, as part of the reward, are predetermined and do not vary by provider. Hence, the reward per patient is also predetermined, and the total payment to a provider is fixed based on the provider’s response in terms of additional patients treated.

The equation applies regardless of whether the P4P scheme targets individual health professionals or teams. Additionally, it is applicable irrespective of the number of patients treated. When there is a reward for each additional patient treated, reward size, treatment costs, and health gains are proportional, and the ICER remains consistent regardless of the number of patients treated and rewarded. That is, treating additional patients for a proportionate reward does not change the ICER. Hence, under an improved or continuous performance standard, calculating the ICER obviates the need to consider the relationship between price and the behavioral response of providers in terms of additional patients treated.

An exception to this general rule may exist in cases where the reward also includes any fixed costs incurred by payers, such as an overhead portion (not received by physicians) for implementing and administering the program at the payer level. A P4P program initiated for one disease is typically an extension of existing P4P programs. Hence, the fixed costs are semi-fixed and may result from additional administrative staffing, hardware, software licenses, or IT system upgrades. If we assume a fixed cost per batch of patients in P4P programs and then allocate it to each patient, this method becomes equivalent to assuming a fixed cost portion for each individual patient, provided that the batch size remains constant and does not vary over time or across different batches. Nevertheless, adjustments to the cost allocation model may be required if significant deviations from the expected batch size are expected. While in the latter case, the behavioral response of providers in terms of additional patients treated matters, the calculation of the semi-fixed cost per patient for a specific batch of patients requires a granularity of data that may not be readily available (e.g., considering that overhead costs vary by type of P4P program or patient population). Therefore, it may be pragmatically sufficient to assume a fixed cost portion for each individual patient. Moreover, considering that administrative costs typically range from 1 to 3% of total program spending for P4P programs, especially in well-established programs with advanced healthcare infrastructure, the impact of including fixed costs in ICER calculations seems relatively small.

Scale effects in P4P programs can lead to the extension of treatments to patients with comparatively lesser direct benefits, due to the limited size of the group most evidently in need. Once treatments for the readily identifiable ‘low-hanging fruit’ patients are administered, healthcare providers are compelled to address the needs of increasingly marginal patients—individuals less likely to see significant health improvements from interventions—to sustain the level of improvement mandated by P4P incentives. McLintock et al. [24] provide real-world evidence of this phenomenon. In light of scale effects, it becomes necessary to compute ICERs for distinct subpopulations, including those considered marginal. This calculation is consistent with the modeling of costs and effects of treatments induced by the P4P program, which is typically done at the cohort or subpopulation level, not at an individual level. It is important to note that these calculations assume additional patients treated are comparable to the population in which treatment costs and health gains were originally estimated. As explained in the previous paragraph, the calculation of ICERs for each subpopulation essentially remains unaffected by their respective sizes.

In contrast to an improved or continuous performance standard, absolute or relative performance mechanisms may not reward providers, even when performance is improved. In this scenario, the calculation of the reward needs to account for the proportion of the patient population under treatment by providers who meet a specific threshold and are thus eligible for rewards. While, in the case of a relative performance scheme, this proportion can be set ex ante, in the case of an absolute performance threshold, it depends on the behavioral response of providers. Denoting *α* as the proportion of the patient population under treatment by providers who pass the performance threshold, we derive from Eq. 2:

3 $$\:ICER=\frac{\alpha\:\times\:n_{\text{P}4\text{P}}\times\:\Delta c_{\text{P}4\text{P}}}{\Delta b_\text{tx}}+\frac{\Delta c_\text{tx}}{\Delta b_\text{tx}}.$$

Of note, $\:\alpha\:$ in Eq. 3 specifically refers to the reward paid to providers who meet the performance threshold (high performers). However, it does not refer to the costs and benefits of treatments induced by the reward, including the costs and benefits of treating additional patients by providers who do not meet the performance threshold (low performers). Equation 3 encompasses the costs and benefits associated with treatments for additional patients treated by providers failing to pass the performance threshold in the second term. Furthermore, the second term, capturing average changes among high and low performers, accounts for any potential decline in care quality from low performers who may give up, offset either partially or fully by improvements from high performers (see Eq. 4 below). The incremental costs and health benefits, represented by $\:\varDelta{c}_{\text{t}\text{x}}$ and $\:\varDelta{b}_{\text{t}\text{x}}$, remain constant across the different performance levels of providers, including those above (denoted by $\:\alpha\:$) and below (denoted by $\:\stackrel{-}{\alpha\:}$) the performance threshold. That is, while the distribution of provider performance affects the allocation of rewards, it does not alter the fundamental cost-effectiveness relationship of the treatment. Providers performing below the threshold are further categorized into positively ($\:\stackrel{-}{{\upalpha\:}},\text{p}\text{o}\text{s}$) and negatively ($\:\stackrel{-}{{\upalpha\:}},\text{n}\text{e}\text{g}$) performing groups, but these distinctions do not impact the overall cost-effectiveness ratio as demonstrated by the following equation:

4 $$\begin{aligned}&\:\frac{n_{\overline\alpha,\text{neg}}\times\:\left(-\Delta c_\text{tx}\right)+\left(n_{\overline\alpha,\;\text{pos}}+n_{\alpha\:}\right)\times\:\Delta c_\text{tx}}{n_{\overline\alpha,\text{neg}}\times\:\left(-{\Delta}b_\text{tx}\right)+\left(n_{\overline\alpha,\text{pos}}+n_{\alpha\:}\right)\times\Delta{b}_\text{tx}}\\&=\frac{\left(n_{{}_{\overline\alpha,\;\text{pos}}}+n_{\alpha\:}-n_{\overline\alpha,\;\text{neg}}\right)\times\:\Delta{c}_\text{tx}}{\left(n_{\overline\alpha\:,\text{pos}}+n_{\alpha\:}-n_{\overline\alpha\:,\text{neg}}\right)\times\:\Delta{b}_\text{tx}}=\frac{\Delta c_\text{tx}}{\Delta{b}_\text{tx}},\end{aligned}$$

5 $$n_{\bar{\mathrm{\alpha}}}=n_{\bar{\mathrm{\alpha}},{\mathrm{neg}}}+n_{\bar{\mathrm{\alpha}},{\mathrm{pos}.}}$$

Here, $n_{\alpha}$ and $n_{\overline{\alpha}}$ refer to the number of patients treated by providers who pass the threshold or not, respectively.

Equation 3 shows that when $\:\alpha\:$ (the proportion of patients treated by high-performing providers) is small, the ICER becomes more favorable.

While the per-patient total reward size (the numerator of the first term in Eq. 3) is endogenous for an absolute performance threshold, it is exogenous under a relative performance mechanism given that the proportion of providers who pass the threshold is also exogenous. For improved and continuous performance thresholds, it is also exogenous given that each (additional) patient treated will be rewarded ($\:\alpha\:=1$). Therefore, without requiring information on the effectiveness of a reward, we can determine an ICER for each reward size in these schemes with exogenous rewards, a priori. For all performance schemes, total costs, including those for the reward, range from treatment costs to infinity (representing an infinitely large reward), meaning the lower bound of P4P program costs is set by the costs of treatment. Similarly, the ICER ranges from treatment ICER to infinity.

#### Budget impact calculation

If the aim is to calculate the budget impact of a P4P program under each performance mechanism discussed so far (i.e., improved, continuous, relative, and absolute), information on the relationship between price and the behavioral response of providers, in terms of how many more patients are treated, is still needed. This relationship is formalized using the income elasticity of supply of health professionals *E*, which is calculated as the percentage change in compliance of health professionals divided by the percentage change in income during the period of effectiveness $\:du$:

6 $$\:E=\frac{\frac{{\lambda}_\text{hp}\:\times\:\Delta_\text{hp}}{1-{\lambda}_\text{hp}}}{\frac{n_{\text{P}4\text{P}}\:\times\:\Delta{c}_{\text{P}4\text{P},\text{hp}}}{du\:\times\:income}},$$

where $\:\varDelta{c}_{\text{P}4\text{P},\text{h}\text{p}}$ is the size of a reward received by health professionals and excludes the overhead costs incurred by payers. The income elasticity of supply depends on the resistance of health professionals regarding some of the treatments and the decreasing marginal utility of income of health professionals. Rearranging Eq. 6 yields an expression for the absolute reduction of the quality deficit:

7 $$\:{\lambda}_\text{hp}\times\:{\Delta\lambda}_\text{hp}=\frac{n_{\text{P}4\text{P}}\times\:\Delta{c}_{\text{P}4\text{P},\text{hp}}}{du\times\:income}\times\:\left(1-{\lambda}_\text{hp}\right)\times E.$$

This expression for the absolute reduction of the quality deficit can be integrated into the calculations of the budget impact of a P4P program, as described in the following sections. When the reward is based on improved performance, the budget impact is calculated as follows:

8 $$\begin{aligned}\:\Delta{c}_\text{pop}=\:&n_\text{pop}\times\:p_\text{dx}\times\:\left[\left(n_{\text{P}4\text{P}}\times\:\Delta{c}_{\text{P}4\text{P}}+\Delta{c}_\text{tx}\right)\right.\\&\left.\times\:{\lambda}_\text{hp}\times\:\Delta{\lambda}_\text{hp}\right],\end{aligned}$$

where $\:\varDelta{c}_{\text{p}\text{o}\text{p}}$ represents population costs and $\:{n}_{\text{p}\text{o}\text{p}}$ is the population size.

When the reward is based on absolute performance, the P4P program rewards not only an improvement in performance in providers who pass the threshold but also their existing performance. Calculating the budget impact in this context necessitates adding the cost of rewarding existing performance. Additionally, a change in costs, invoked by those providers who do not pass the threshold but have responded either positively or negatively to it, needs to be considered:

9 $$\begin{aligned} \:\Delta{c}_\text{pop}=\:&n_\text{pop}\times\:p_\text{dx}\times\:\{\alpha\:\times\:\left[\left(n_{\text{P}4\text{P}}\times\:\Delta{c}_{\text{P}4\text{P}}+\Delta{c}_\text{tx}\right)\right.\\&\left.\times\:{\lambda}_{\text{hp},\alpha\:}\times\:\Delta{\lambda}_{\text{hp},\alpha\:}+\left(n_{\text{P}4\text{P}}\times\:\Delta{c}_{\text{P}4\text{P}}\right)\right.\\&\left.\times\:\left(1-{\lambda}_{\text{hp},\alpha\:}\right)\right]+\left(1-\alpha\:\right)\times\:{\lambda}_{\text{hp},\overset-{\alpha\:}}\times\:\triangle{\lambda}_{\text{hp},\overset-{\alpha\:}}\times\Delta{c}_\text{tx}\}\\&=\:n_\text{pop}\times\:p_\text{dx}\times\:\left\{\alpha\:\times\:\left[\left(n_{\text{P}4\text{P}}\times\Delta{c}_{\text{P}4\text{P}}\times\:{\lambda}_{\text{hp},\alpha\:}\right.\right.\right.\\&\left.\left.\left.\times\:\Delta{\lambda}_{\text{hp},\alpha\:}\right)+\left(n_{\text{P}4\text{P}}\times\:\Delta{c}_{\text{P}4\text{P}}\right)\times\:\left(1-{\lambda}_{\text{hp},\alpha\:}\right)\right]\right.\\&\left.+{\lambda}_\text{hp}\times\:\Delta{\lambda}_\text{hp}\times\:\Delta{c}_\text{tx}\right\},\end{aligned}$$

where $\:\alpha\:$ and $\:\stackrel{-}{\alpha\:}$ indicate the proportion of providers who pass the threshold or not, respectively. When the payment is based on relative performance, this calculation also holds and $\:\alpha\:$ refers to those providers who ‘win the tournament’. Furthermore, for a continuous performance scheme, the same equation is used but $\:\alpha\:$ is set equal to 1.

In summary, when a P4P program does not lead to a substitution of other health care programs, its ICER is deterministically determined and depends on the behavioral response of providers mainly in the case of an absolute performance threshold (whereas for the other three schemes discussed the ICER can be calculated a priori). In contrast, the calculation of the budget impact of a P4P program always requires information on the behavioral response of providers.

### P4P substitution model

Now, consider a scenario wherein both a combined budget constraint for financial incentives and treatments, and a time constraint for health professionals, exist. Should the P4P program neither save costs nor remain cost-neutral, it necessitates the substitution of other programs. In this latter scenario, decision-makers ought to set the financial incentive above that of one or more other P4P programs targeted for substitution (for example, within the same therapeutic area), while maintaining the reward plus treatment ICER beneath that of the P4P programs being substituted. This is crucial to ensure that population health is not diminished, all other factors held constant (ceteris paribus).

The budget impact and incremental population health benefits are computed as the differential in population costs and health benefits between the P4P program and one or more programs that it replaces. When a P4P program establishes a reward based on improved performance and partially substitutes another program, the incremental population costs and health benefits are calculated as follows:

10 $$\begin{aligned}\Delta{c}_{\mathrm{pop}}=\:&n_{\mathrm{pop}}\times\:\left[{\left(p_{\mathrm{dx}}\times\:\left(n_{\mathrm P4\mathrm P}\times\:\Delta{c}_{\mathrm P4\mathrm P}+\Delta{c}_{\mathrm{tx}}\right)\times\:\lambda_{\mathrm{hp}}\times\:{\Delta\lambda}_{\mathrm{hp}}\right)}_{\mathrm A-\mathrm B}\right. \\&\left.-{\left(p_{\mathrm{dx}}\times\:\Delta{c}_{\mathrm{tx}}\times\:\lambda_{\mathrm{hp}}\times\:{\Delta\lambda}_{\mathrm{hp}}\right)}_{\mathrm C-\mathrm D}\right],\end{aligned}$$

11 $$\begin{aligned}\Delta{b}_{\text{p}\text{o}\text{p}}=\:&{n}_{\text{p}\text{o}\text{p}}\times\:\left[{\left({p}_{\text{d}\text{x}}\times\:{{\lambda}}_{\text{h}\text{p}}\times\:{\Delta{\lambda}}_{\text{h}\text{p}}\times\:\Delta{b}_{\text{t}\text{x}}\right)}_{\text{A}-\text{B}}\right.\\&\left.-{\left({p}_{\text{d}\text{x}}\times\:{{\lambda}}_{\text{h}\text{p}}\times\:{\Delta{\lambda}}_{\text{h}\text{p}}\times\:\Delta{b}_{\text{t}\text{x}}\right)}_{\text{C}-\text{D}}\right].\end{aligned}$$

Here, A and B represent the P4P program under scrutiny and its comparator, while A-B illustrates the incremental difference between A and B. Similarly, C and D denote the P4P program set for replacement and its comparator, and C-D illustrates the incremental difference between C and D. Note that $\:\varDelta{b}_{\text{p}\text{o}\text{p}}$ symbolizes incremental population health benefits. The implementation of program C, whether through education, financial incentives, contracts, or other means, may incur costs (not explicitly shown). Without incentives for program C, there is a disincentive for its continuation, leading to a decrease in physician compliance with program C as compared to D.

Under a budget constraint, it is imperative that $\:\varDelta{c}_{\text{p}\text{o}\text{p}}$ remains non-positive. Consequently, the budget holder must configure the reward of program A such that

12 $$\begin{aligned}\:&{\left({p}_{\text{d}\text{x}}\times\:\left({n}_{\text{P}4\text{P}}\times\:\Delta{c}_{\text{P}4\text{P}}+\Delta{c}_{\text{t}\text{x}}\right)\times\:{{\lambda}}_{\text{h}\text{p}}\times\:{\Delta{\lambda}}_{\text{h}\text{p}}\right)}_{\text{A}-\text{B}}\\&\le\:{\left({p}_{\text{d}\text{x}}\times\:\Delta{c}_{\text{t}\text{x}}\times\:{{\lambda}}_{\text{h}\text{p}}\times\:{\Delta{\lambda}}_{\text{h}\text{p}}\right)}_{\text{C}-\text{D}},\end{aligned}$$

while simultaneously ensuring that the reward plus treatment ICER of program A is inferior to that of program C. Notably, the reward for program A can only be determined ex ante based on Eq. 12 if programs A and C are substitutes. However, if they are not substitutes or the substituted program is not identified (indicating an absence of a priori information about the health professionals’ behavioral response), then the reward size will need to be set ex post, even though a reward is being announced ex ante.

Moreover, in instances where the program to be replaced has relatively low treatment costs ($\:\varDelta{c}_{\text{t}\text{x}}$) despite a high ICER, or where it targets a small population (thus, the value of $\:{p}_{\text{d}\text{x}}$ is small), any reward size might drive $\:\varDelta{c}_{\text{p}\text{o}\text{p}}$ to a positive value. This can also occur when the financial incentive for A triggers substitution not just of program C (which has a higher ICER than A) but also of programs with a reduced ICER if the incentive for these interventions is lower. When substituting the latter, it is plausible to encounter the unfavorable outcome wherein introducing A results in escalated costs and/or diminished health benefits. An example is provided in the appendix.

It is important to note that the existence of a larger incentive for A than for C does not inherently ensure that A will be implemented. This is particularly true if providers, whether correctly or incorrectly, presume that C confers greater health benefits than A and their altruistic motivations surpass their utility derived from additional income. Moreover, a substantial incentive may not induce substitution if the intervention necessitates a disproportionally large allocation of a professional’s time, thereby accruing higher costs per minute of a professional’s engagement. Hence, to be precise, the incentive must be larger on a per-patient, per-minute basis.

When the reward is grounded in absolute performance, the budget holder must structure the incentive in accordance with Eq. 9, ensuring that:

13 $$\begin{aligned}&\Big({p}_{\text{d}\text{x}}\times\:\Big\{\alpha\:\times\:[\left({n}_{\text{P}4\text{P}}\times\:\Delta{c}_{\text{P}4\text{P}}+\Delta{c}_{\text{t}\text{x}}\right)\\&\times\:{{\lambda}}_{\text{h}\text{p},{\alpha\:}}\times\:{\Delta{\lambda}}_{\text{h}\text{p},{\alpha\:}}+\left({n}_{\text{P}4\text{P}}\times\:\Delta{c}_{\text{P}4\text{P}}\right)\\&\times\:\left(1-{{\lambda}}_{\text{h}\text{p},{\alpha\:}}\right)]+\left(1-\alpha\:\right)\times\:{{\lambda}}_{\text{h}\text{p},\stackrel{-}{{\alpha\:}}}\\&\times\:{\Delta{\lambda}}_{\text{h}\text{p},\stackrel{-}{{\alpha\:}}}\times\:\Delta{c}_{\text{t}\text{x}}\Big\}\Big)_{\text{A}-\text{B}}\\&\le\:{\left({p}_{\text{d}\text{x}}\times\:\Delta{c}_{\text{t}\text{x}}\times\:{{\lambda}}_{\text{h}\text{p}}\times\:{\Delta{\lambda}}_{\text{h}\text{p}}\right)}_{\text{C}-\text{D}},\end{aligned}$$

wherein program C remains non-incentivized to facilitate its replacement. Additionally, the incentive is constrained by the reward plus treatment ICER of program A, which must persist below that of program C to enhance population health. In scenarios where payment is contingent upon relative or continuous performance, the calculations adhere to the same paradigm as for an absolute performance scheme. Once again, $\:\alpha\:$ refers to providers who ‘win the tournament’ (in the context of a relative performance scheme) or is set equal to 1 (in scenarios pertaining to a continuous improvement scheme).

### Case studies of the P4P model

This section presents hypothetical case studies to examine the potential impact of two types of P4P programs—focused on improved and absolute performance rewards—within a target population of 1,000,000 patients. The case studies are predicated on a disease prevalence ($\:{p}_{\text{d}\text{x}}$) of 10% within the target population.

#### Improved performance reward

Leveraging Eqs. 10 and 11, we explored the potential outcomes of a hypothetical P4P program A, which incentivizes improved performance, alongside a comparison with a substitute program C. We posited that 50% of this population was affected by deficits in the quality of care, with program A achieving a 20% relative improvement in this area.

We quantified the per-patient health benefits in terms of quality-adjusted life years (QALYs), assuming enhancements of 0.2 QALYs for program A and 0.1 QALYs for program C per treated patient. The treatment costs were delineated as €200 for program A and €1,800 for program C per patient. Furthermore, we modeled three P4P payments ($\:{n}_{\text{P}4\text{P}}$) for each patient undergoing treatment, with each P4P payment ($\:\varDelta{c}_{\text{P}4\text{P}}$) priced at €500. This figure encapsulates the aggregate expenses associated with incentive disbursements and administrative operations.

The application of these parameters to our model (Eqs. 10 and 11) disclosed an incremental population savings ($\:\varDelta{c}_{\text{p}\text{o}\text{p}}$) of €1 million and an augmented health benefit ($\:\varDelta{b}_{\text{p}\text{o}\text{p}}$) of 1,000 QALYs for the population under study.

#### Absolute performance reward

In this scenario, a bonus is contingent upon a provider’s performance exceeding a predetermined benchmark—a 60% compliance rate among physicians. We assumed that 20% of the patient cohort received treatment from providers meeting this performance threshold (*α*). The average baseline compliance rate of physicians above and below the target standard was set at 70% and 30%, respectively. Program A was assumed to achieve a 20% relative improvement in physicians both above and below the target standard.

By applying these inputs to Eq. 13 for the calculation of both population savings and health benefits, we calculated an incremental population savings ($\:\varDelta{c}_{\text{p}\text{o}\text{p}}$) of €3,280,000 and a health benefit increment ($\:\varDelta{b}_{\text{p}\text{o}\text{p}}$) of 1,480 QALYs for the million-strong population. Per Eq. 13, the analysis accounts for improvements in the management of patients under treatment by providers that do not meet the threshold for incentive payments.

The outcomes from the case studies exemplify the model’s application in evaluating the cost-effectiveness of P4P programs, emphasizing the financial viability of investing in quality improvements.

## Discussion

Utilizing decision modeling, this study illustrates that calculating the ICER for P4P programs—with improved, continuous, or relative performance standards—within the realm of conventional cost-effectiveness analysis (i.e., disregarding time and budget constraints) does not necessitate information regarding the effectiveness of P4P. This contradicts the prevalent belief that an empirical estimate for the effectiveness of a P4P program, potentially derived from a trial, is invariably required. 

The model, as outlined by Eqs. 2 and 3 and characterized by a lack of substitution effects, indicates that less than 20% of P4P programs aimed at addressing underuse of services are likely to be cost-saving. This conclusion is independent of both the structure of the incentive scheme and the size of the incentives offered. This assertion is supported by a review of past literature, which reveals that only approximately 20% of cost-utility analyses report savings [25]. This is explained by the fact that the minimum cost associated with P4P programs is defined by the costs of the treatments being incentivized. Therefore, P4P programs cannot generate cost savings beyond those derived from the efficiencies of the treatment itself. Highlighting this finding is crucial, especially in light of the current literature’s ongoing debate about the cost-saving potential of P4P programs.

However, when budget and time constraints are imposed, the substitution of other programs often becomes inevitable, and the resulting impact on costs and health benefits cannot be predetermined. Specifically, the budget and health impact depend on the size and number of programs being replaced. This study identifies circumstances where a P4P program can lead to increased costs and reduced health benefits under budget or time constraints. These inefficiencies may arise when substituted programs have lower financial incentives but greater cost-effectiveness, further diminishing the potential savings of P4P programs.

The decision model presented in this paper has certain limitations, particularly in not fully accounting for potential negative repercussions of P4P strategies. Snyder et al. [26] outline four key challenges in a review article: firstly, the difficulty of achieving perfect risk adjustment may incentivize providers to avoid more severe cases, a practice known as ‘cherry-picking’; secondly, focusing on specific metrics may result in the deterioration of unmeasured aspects of care, known as ‘playing to the measures’; thirdly, enforcing uniform quality standards across all patients may inadvertently increase unnecessary care and associated medical costs; and fourthly, P4P programs may erode patient-physician trust. Additionally, there is a risk that providers may misreport health outcomes to increase reimbursement or avoid penalties [27]. Conversely, P4P programs can produce positive spillover effects; for example, incentivizing a specific service like cervical cancer screening might inadvertently improve other unrewarded services, such as chlamydia screening [28]. While the decision model partially addresses the second issue by modeling substitution effects, the other aspects warrant further exploration and inclusion. Notably, incentives for ‘cherry-picking’ are reduced by linking rewards to treatments rather than outcomes. Future research on the cost-effectiveness and economic impact of P4P programs should consider integrating these factors.

## Supplementary Information

Supplementary Material 1.

## Data Availability

All data are contained within the manuscript.
