# Supplementary material for: Modelling the cost-effectiveness and budget impact of pay-for-performance programs in health care
Source: BMC Health Serv Res. 2024 Nov 27;24:1484. doi: 10.1186/s12913-024-11796-1 (PMC11603998; doi:10.1186/s12913-024-11796-1)
Supplement: Supplementary file 1 — Supplementary Material 1. [file 12913_2024_11796_MOESM1_ESM.docx]

**Appendix: Application example of Pay-for-Performance Programs**

This appendix presents a case study that explores how reward sizes and the substitution between interventions can impact overall costs and health outcomes. It highlights that, while financial rewards are designed to improve cost-effectiveness, unintended consequences—such as increased costs without proportionate improvements in health benefits—may occur. To illustrate this, consider two interventions, labeled A and C, each with distinct treatment costs and associated health benefits, as shown below:

Table A1

|  | $\Delta c_{\mathrm{tx}}$ (€) | $\Delta b_{\mathrm{tx}}$ | $\Delta c_{\mathrm{tx}}$/$\Delta b_{\mathrm{tx}}$ (€) |
| --- | --- | --- | --- |
| Intervention A | 1000 | 0.1 | 10,000 |
| Intervention C | 2000 | 0.1 | 20,000 |

Utilizing Equation 2, we can compute the reward plus treatment incremental cost-effectiveness ratios (ICERs) for both interventions, contingent upon the reward size. The corresponding ICER functions are depicted in Figure A 1 below. Assuming the reward size for intervention C is infinitesimally larger than for A, substitution leads to a cost increase while maintaining consistent health benefits.

Figure A1. Cost-effectiveness of pay-for-performance programs.


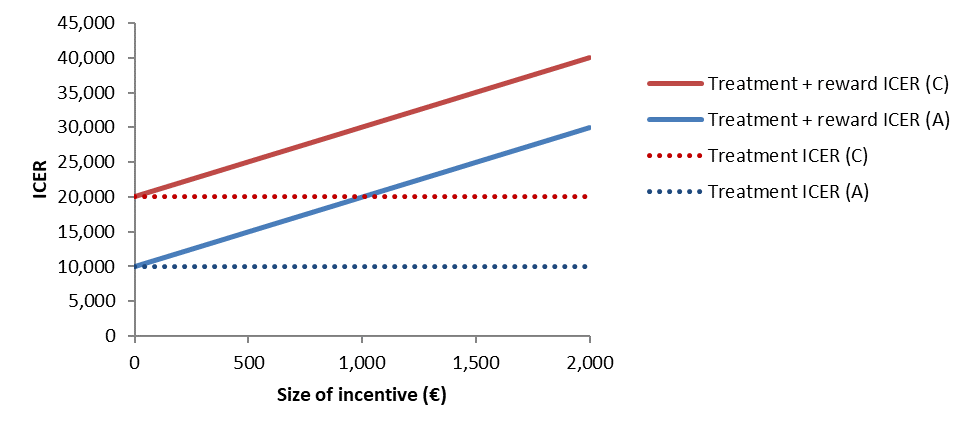


ICER, incremental cost-effectiveness ratio

**Reading Grid**

- X-axis: Reward size (€) provided for pay-for-performance programs.
- Y-axis: Adjusted incremental cost-effectiveness ratio (ICER in €/QALY).
- The solid and dashed lines represent the ICER values for Intervention A and Intervention C, with and without the reward incentive. The ICER for each intervention changes as the size of the financial incentive increases.

**Legend**

- Solid Red Line: Treatment + reward ICER (Intervention C)
- Solid Blue Line: Treatment + reward ICER (Intervention A)
- Dashed Red Line: Treatment ICER (Intervention C)
- Dashed Blue Line: Treatment ICER (Intervention A)
